# Effect of breathing exercises on depression, sexual function, and exercise capacity in postmenopausal women: a randomized controlled trial

**DOI:** 10.1038/s41598-026-41198-8

**Published:** 2026-02-23

**Authors:** Saher Lotfy Elgayar

**Affiliations:** Department of Physiotherapy, Faculty of Allied Medical Sciences, Middle East University, Amman, Jordan

**Keywords:** Menopause, Women, Depression, Breathing exercises, Sexual function, Diseases, Health care, Medical research

## Abstract

This trial aimed to explore the effect of supervised breathing exercises (BEs) on depression, sexual function, and exercise capacity in postmenopausal women. Sixty-four depressed postmenopausal women with associated sexual dysfunction were equally assigned to either a BEs group, which received diaphragmatic BEs, or a non-exercising control group. Both groups received individualized antidepressants and vaginal lubricants. Depressive symptoms were assessed using the Beck Depression Inventory-II (BDI-II), sexual function using the Female Sexual Function Index (FSFI), and exercise capacity using the six-minute walk test (6MWT) at baseline and after 12 weeks. At the end of the study, the BEs group showed significantly greater reductions in BDI-II scores (mean difference = − 4.22; 95% CI, − 6.19 to − 2.24; *p* = 0.001) and significantly greater increases in FSFI scores (mean difference = 5.01; 95% CI, 3.21 to 6.79; *p* = 0.001) compared with the control group with clinically significant differences for both measures. However, the between-group difference in 6MWT distance was not significant (mean difference = 28.28 m; 95% CI, − 2 to 58.57; *p* = 0.06). BEs could be an effective therapeutic intervention for managing depression and sexual dysfunction in this population of postmenopausal women, while no significant effect was observed on exercise capacity.

**Supplementary Information:**

The online version contains supplementary material available at 10.1038/s41598-026-41198-8.

## Introduction

Menopause is a transitional phase marked by ovarian failure and hormonal, physical, and emotional changes that affect psychological well-being^1^ and sexual function^2^. These changes are often associated with a reduced quality of life^3^. Depression affects 34.9% of postmenopausal women worldwide^4^, primarily due to hormonal changes that impact hypothalamic and hippocampal brain function^5^, as well as the negative effects of vasomotor symptoms such as hot flushes^6^ and sleep disturbances^7^. Sexual function often declines after menopause due to discomfort during sexual intercourse^8^. The decline in gonadal steroid hormones contributes to decreased vaginal lubrication and elasticity, often resulting in vaginal atrophy and painful intercourse^9^. Sexual dissatisfaction may lead to insecurity, frustration, and a sense of inadequacy^10^, further lowering quality of life^11^. Additionally, women after menopause exhibit lower exercise capacity levels than pre-menopausal ones^12^.

In different populations, breathing exercises (BEs) have been shown to enhance parasympathetic activity and reduce cortisol levels^13,14^, mechanisms that may alleviate depressive symptoms. BEs can reduce pelvic muscle tension^15^, which may positively influence sexual function, and improve respiratory efficiency and oxygen delivery^16^, supporting exercise tolerance and functional capacity. In postmenopausal women, BEs have gained attention as accessible, non-pharmacological interventions that may improve both physical and mental health outcomes^17–20^. In such women, BEs have been shown to enhance cognitive performance and postural control^17,18^, reduce hot flushes and improve quality of life^19^, improve respiratory function^18^, and enhance sleep quality^20^. However, despite the promising benefits of BEs on these health domains in postmenopausal women^17–20^, to the best of our knowledge, no published randomized controlled trial has specifically investigated the combined effects of BEs on depression, sexual function, and exercise capacity in this population. This identified gap led to the present randomized controlled trial, which was designed to evaluate the impact of a structured BEs regimen on depression as the primary outcome, and on sexual function and exercise capacity as secondary outcomes among postmenopausal women.

## Materials and methods

### Study settings and ethical principles

This study followed the guidelines of the CONSORT 2010 statement^21^ and strictly adhered to the principles outlined in the Declaration of Helsinki. Between 26 December 2024 and 30 May 2025, this prospective, randomized controlled, parallel-group trial was conducted as a multicenter study, recruiting women equally from three hospitals located in different governorates in Egypt: Samanoud Public Hospital (Gharbia), Mansoura General Hospital (Dakahlia), and Kafr Alsheikh General Hospital (Kafr Alsheikh). Participants were recruited from the gynecology outpatient clinics at these sites through direct referrals by gynecologists. All sites followed the same standardized protocol, with uniform training for staff and centralized monitoring of procedures and data collection, to ensure consistency across centers. Following ethical approval from the Ethics Committee for Human Scientific Research at Cairo University (Approval No: P.T.REC/012/005385), a total of 172 postmenopausal women were initially screened. Of these, only 71 women (41.27%) met the inclusion criteria. After seven women declined to participate, 64 participants were ultimately enrolled in the study. All participants provided written informed consent before the initiation of the intervention, which was conducted in a private physical therapy unit in Egypt. With the assigned number of NCT06741384, the study has been registered prospectively on 18 December 2024 through clinicaltrials.gov available at: https://clinicaltrials.gov/study/NCT06741384.

### Sample size estimation

The required sample size for each group was calculated using G*Power version 3.1 software. The calculation was based on the Beck Depression Inventory-II (BDI-II) score as the primary outcome measure, using post-intervention data from the study of Chung et al.^22^, which resulted in an effect size of 1.35. A two-sided independent samples t-test was employed with a 5% significance level (α) and 95% statistical power. Based on these parameters, the required sample size was calculated to be 16 participants per group. However, to further increase statistical power, enhance the reliability of the results, and ensure robustness against potential dropouts, the final sample size was doubled to 32 participants per group, yielding a total of 64 postmenopausal women.

### Randomization, allocation, and blinding

Participants were randomly assigned to the study and control groups using simple randomization with computer-generated random numbers (Stata 17, StataCorp LLC, USA), maintaining a 1:1 allocation ratio. To maintain allocation concealment, sequentially numbered, opaque, sealed envelopes were employed and opened solely after participants had been enrolled. Recruitment was conducted by two physiotherapists, while group assignment was performed by a gynecologist. Both the recruiters and the assigner were not involved in the study and were blinded to group allocations. Outcome assessors and the physician prescribing medications were blinded to group assignments throughout the study and until post-intervention assessments were completed. The outcome assessors did not participate in administering the exercise intervention. However, due to practical constraints, blinding of participants and exercise supervisors was not feasible.

### Participants

Sixty-four postmenopausal, married women with the following inclusion criteria were considered for the study: (a) natural menopause confirmed clinically by the absence of menstruation for at least 12 consecutive months^23^ with more than three years since the onset of menopause; (b) mild to moderate depressive symptoms, indicated by BDI-II scores between 14 and 28^24^; (c) mild to moderate sexual dysfunction, based on the Female Sexual Function Index (FSFI), with scores between 7.3 and 28.1^25^; (d) regular vaginal sexual intercourse, at least once per week; (e) age between 48 and 60 years, with a body mass index of less than 30 kg/m². Exclusion criteria included premature menopause, psychiatric disorders other than depression, pelvic organ prolapse, vaginal infections, diabetes, and contraindications to exercise testing, including unstable cardiovascular conditions, severe respiratory diseases, musculoskeletal disorders that limit exercise participation, neurological disorders affecting balance or motor function, and any other medical condition deemed to render exercise unsafe. Recruitment continued until the target sample size of 64 participants was reached. The participants were then randomly allocated into two equal groups: the BEs group, which received diaphragmatic BEs in addition to medical treatment, and a control group, which received medical treatment only, with no exercise intervention.

### Evaluations

#### History and clinical assessment

A highly experienced gynecologist was responsible for confirming participants’ eligibility through a comprehensive assessment. This included a detailed review of age, race, duration since menopause, and obstetric history such as number and types of deliveries. Current medications, associated medical conditions, and the participants’ current level of physical activity, assessed using the validated six-point physical activity scale^26^, were also screened. Each participant underwent a thorough clinical examination. Additionally, baseline anthropometric measurements, including weight and height, were assessed twice for all participants using an electronic scale (Model: BYH01, China), and the average of the two measurements was used to calculate body mass index.

#### Depression (primary outcome)

To assess the level of depressive symptoms, all participants in both groups were asked to independently complete the Arabic validated version of the BDI-II^27^ during personal interviews, both at baseline and post-intervention. The BDI-II comprises 21 questions, each rated on a 0 to 3 scale, producing a total score between 0 and 63, where higher scores reflect greater severity of depressive symptoms. A total BDI-II score of 14 or higher is considered indicative of depression, with scores of 14–19 reflecting mild depression, 20–28 moderate, 29–36 severe, and over 36 extremely severe depression^24^.

#### Sexual function (secondary outcome)

This research utilized the Arabic version of the FSFI, validated by Anis et al.^28^, to assess sexual function at baseline and after twelve weeks, with all questionnaires completed independently by participants during face-to-face meetings. The FSFI is composed of 19 individual items that evaluate six dimensions of female sexual function: desire, satisfaction, arousal, lubrication, pain, and orgasm. It yields both domain-specific scores and a total score reflecting overall sexual function. Each item is scored between 0 and 5, where greater scores denote better sexual function^29^. A total FSFI score of 28.2 or lower is considered indicative of sexual dysfunction, categorized as mild (21.7 to 28.1), mild to moderate (14.5 to 21.6), moderate (7.3 to 14.4), and severe (2 to 7.2)^25^.

#### Exercise capacity (secondary outcome)

The six-minute walk test (6MWT) was used to assess exercise capacity in all participants, being a valid and reliable test measure^30^. The protocol followed the American Thoracic Society guidelines^31^. Each participant rested for 10 min before testing, during which the procedure was explained. Two cones placed 30 m apart in a long corridor marked the walking course. Participants were instructed to walk back and forth between the cones for six minutes, covering the greatest possible distance. Standardized encouragement was given every minute, and participants were monitored for any signs requiring test termination. The total distance walked was recorded in meters.

### Interventions

#### Pharmacological treatment

All postmenopausal women in this study received pharmacological treatment with antidepressant medications and vaginal lubricants that were individualized according to each participant’s clinical needs. Antidepressants were prescribed by an independent neurologist and included selective serotonin reuptake inhibitors, serotonin-norepinephrine reuptake inhibitors, or other agents, administered at stable doses. Vaginal lubricants used included water-based, silicone-based, and oil-based products, with comparable distribution between groups. Adherence to antidepressant and lubricant use was confirmed at baseline and monitored throughout the trial by self-report and pill counts during follow-up visits; no changes in medication type or dosage were permitted during the intervention period. This ensured that any differences observed between groups reflected the impact of breathing exercises rather than pharmacological variability. Additionally, a subset of participants in both groups (*n* = 13 per group) were on systemic estrogen-based hormone therapy, which remained unchanged during the study.

#### Breathing exercises (BEs)

For a duration of 12 weeks, women in the BEs group participated in three supervised sessions per week of diaphragmatic BEs, overseen by two physiotherapists with 10 years of experience. The sessions took place in the morning in groups of eight participants, all wearing loose, comfortable clothing. While in a crook-lying position with relaxed shoulders, participants were guided to take a deep, full breath through the nose over three seconds, hold it for three seconds, and then exhale slowly through pursed lips for six seconds^32^. Throughout the study, participants progressed to performing 3 to 5 sets of 5 to 10 repetitions of diaphragmatic BEs, with a two-minute rest between sets. Adherence was tracked beyond session attendance. The exercise supervisors utilized a standardized checklist to document the quality and progression of the diaphragmatic BEs for each participant during every supervised session.

### Analysis of data

The SPSS software program (version 22; SPSS Inc., Chicago, IL) was used for statistical analysis. Data were first screened for outliers and assessed for normality using the Shapiro–Wilk test (*p* > 0.05). Continuous variables were presented as means and standard deviations, whereas frequencies and percentages described categorical variables. Changes within each group were assessed using the paired t-test, while differences in change scores (post–pre) between groups were analyzed using the unpaired t-test. Between-group differences were expressed as mean difference (MD) with corresponding 95% confidence interval (95% CI). Also, Cohen’s effect size (d) was computed as the mean difference in change scores between groups divided by the pooled standard deviation and interpreted as small (0.2), medium (0.5), and large (0.8)^33^. The chi-square test was employed to compare baseline categorical variables. A significance level of 0.05 was established for all statistical analyses. All statistical analyses were performed using a per-protocol approach, including only participants who completed baseline and post-intervention assessments. Participants with missing post-intervention data were excluded from the analysis, and no imputation was performed.

## Results

**Fig. 1:**
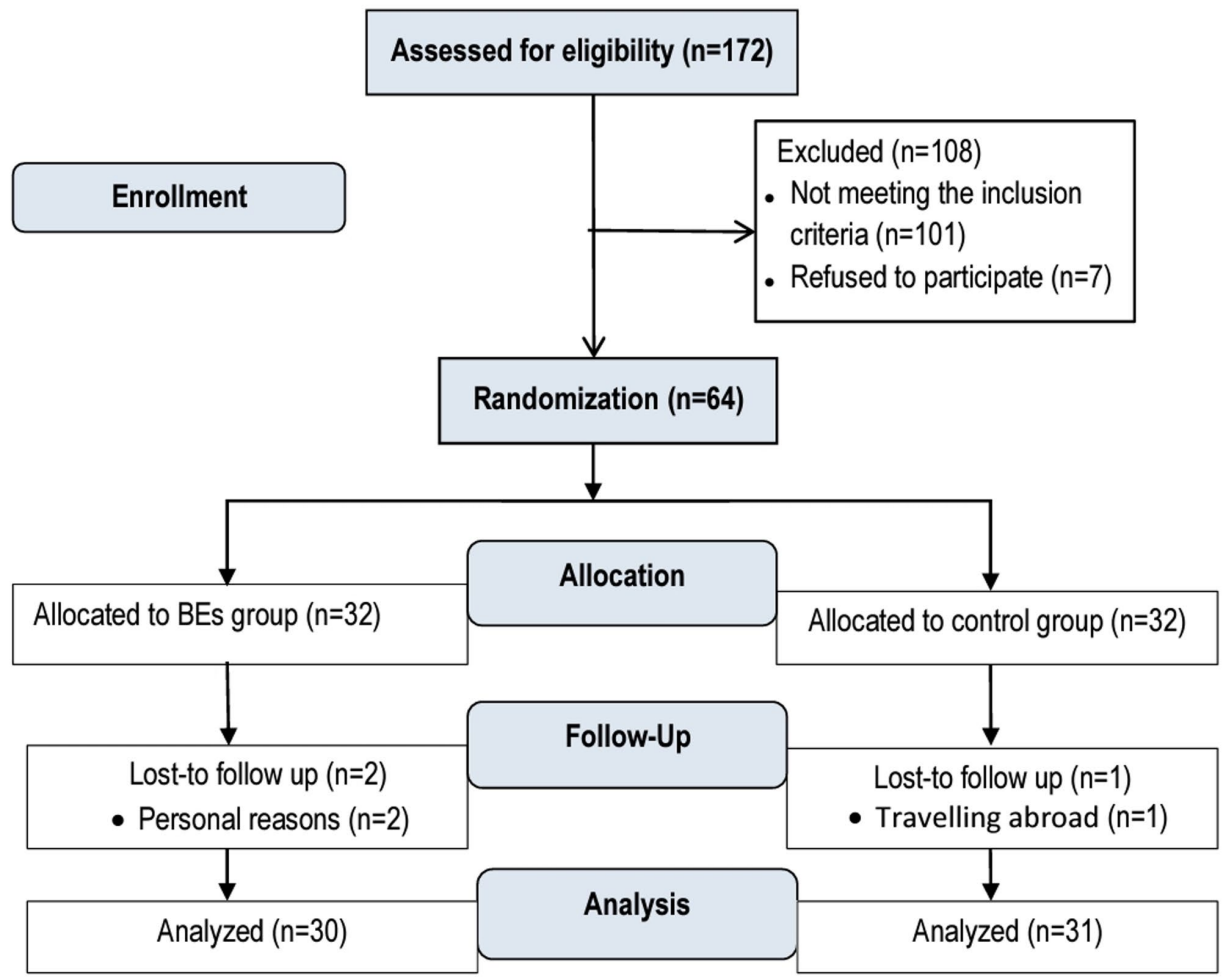
Phases of the trial.

The participant flow throughout the trial is detailed in the CONSORT flow diagram (Fig. 1). During the study, two participants from the BEs group withdrew from the study due to personal causes, and one withdrew from the control group due to traveling abroad. At post-study, the adherence rate in the BEs group was 91.66%, calculated based on the total number of training sessions attended relative to the total planned sessions, with a mean attendance of 33 ± 0.93 sessions per participant. This high adherence was maintained through continuous follow-up and feedback from the supervisors. Regarding exercise quality, supervisor checklists indicated that 96.66% of women in the BEs group achieved proficiency in diaphragmatic breathing technique within the first three sessions. Adherence remained consistent over the course of the intervention, with no apparent decline in attendance or performance quality observed between the initial and final weeks of the program. No adverse effects related to the interventions were reported throughout the study. A comparison of demographic, anthropometric, and clinical characteristics between the two groups revealed no significant differences at baseline (*p* > 0.05) (Table 1). The types and doses of medications used by participants were similar between the two groups at baseline, with no significant differences observed (*p* > 0.05). Similarly, baseline scores for the BDI-II and FSFI revealed no statistically significant differences across groups (*p* > 0.05) (Table 2).

**Table 1:** Women’s baseline characteristics. Data is expressed using means with standard deviations and frequencies. Unpaired t-test compared continuous variables and chi-square test compared categorical ones. A p-value < 0.05 is significant. SSRIs: Selective serotonin reuptake inhibitors, SNRIs: Serotonin and norepinephrine reuptake inhibitors, and BEs: breathing exercises.

Variable		BEs (*n* = 30)	Control (*n* = 31)	*p*-value
Age (years)		51.93 ± 4.34	53.41 ± 3.93	0.16
Weight (kg)		71.5 ± 9.04	72.77 ± 8.11	0.56
Height (cm)		164.9 ± 5.17	166.25 ± 5.69	0.33
Body mass index (kg/m^2^)		26.55 ± 2.7	25.88 ± 2.81	0.35
Race	White	23 (76.6%)	25 (80.64%)	0.7
	Black	7 (23.3%)	6 (19.35%)	
Employment status	House wife	12 (40%)	9 (29.03%)	0.59
	Employed	15 (50%)	17 (54.83%)	
	Retired	3 (10%)	5 (16.12%)	
Educational level	Primary school	9 (30%)	3 (9.67%)	0.12
	Secondary school	14 (46.6%)	20 (64.51%)	
	University	7 (23.3%)	8 (25.8%)	
Postmenopausal duration (years)		7.06 ± 1.89	6.67 ± 2.05	0.44
Obstetrical history	Number of pregnancies	3.03 ± 1.15	2.61 ± 1.74	0.27
	Number of vaginal deliveries	1.73 ± 0.73	1.87 ± 0.84	0.5
	Children count	2.56 ± 1.19	2.54 ± 1.74	0.96
Estradiol hormone (pg/mL)		16.21 ± 2.3	15.02 ± 2.27	0.08
Antidepressant medications	SSRIs	11 (36.6%)	12 (38.7%)	0.93
	SNRIs	10 (33.3%)	9 (29%)	
	Other	9 (30%)	10 (32.2%)	
Vaginal lubricants	Water-based	9 (30%)	6 (19.35%)	0.6
	Silicone-based	11 (36.6%)	14 (45.16%)	
	Oil-based	10 (33.3%)	11 (35.48%)	
Estrogen hormonal therapy		13 (43.3%)	13 (41.93%)	0.91
6-point physical activity scale		2.43 ± 1.04	2.32 ± 0.9	0.65

**Table 2:** Results of outcome measures. Means and standard deviations are reported; a p-value lower than 0.05 denotes statistical significance. *denotes significant paired t-test test. **denotes significant unpaired t-test . BEs stands for breathing exercises, MD for mean difference, CI for confidence interval, Cohen’s d for Cohen’s effect size, BDI-II for the 2nd edition of Beck Depression Inventory, FSFI stands for Female Sexual Function Index, and 6MWTfor six-minute walk test.

Outcome		BEs (*n* = 30)	Control (*n* = 31)	MD (95% CI)	Cohen’s d	*p*-value
BDI-II	Pre-study	19.43 ± 4.48	19.32 ± 4.15			0.92
	Post-study	11.6 ± 4.71*	15.7 ± 4.78*			
	Mean change	-7.83 ± 4.6	-3.61 ± 2.94	-4.22 (-6.19, -2.24)	1.1	0.001**
FSFI total score	Pre-study	18.77 ± 1.68	19.1 ± 2.13			0.5
	Post-study	26.72 ± 3.56*	22.05 ± 3.01*			
	Mean change	7.95 ± 3.7	2.94 ± 3.28	5.01 (3.21, 6.79)	1.43	0.001**
6MWT (m)	Pre-study	402 ± 54.94	385.16 ± 54.61			0.23
	Post-study	444.8 ± 43.76	399.67 ± 32.54			
	Mean change	42.8 ± 60.54*	14.51 ± 57.67	28.28 (-2, 58.57)	0.48	0.06

### Depression

At baseline, 66.6% of the women in the BEs group had mild depression and 33.3% had moderate depression, compared to 61.29% and 38.87%, respectively, in the control group. These differences were not statistically significant, as determined by the chi-square test (*p* = 0.86). At post-intervention, 70% of the women in the BEs group achieved a BDI-II score below 14, compared to only 9.6% in the control group, reflecting significant reductions in depression scores in both groups (*p* < 0.05) (Table 2). Additionally, the BEs intervention resulted in significantly greater reductions in BDI-II scores compared to the control group (MD = − 4.22, 95% CI = − 6.19 to − 2.24, *p* = 0.001), representing a large effect size (Cohen’s d = 1.1) (Table 2).

### Sexual function

At baseline, 53.33% of the women in the BEs group had mild sexual dysfunction and 46.66% had moderate dysfunction, compared to 54.83% and 45.16%, respectively, in the control group. These differences were not statistically significant, as determined by the chi-square test (*p* = 0.97). At post-intervention, both groups showed significant within-group improvements in FSFI total scores (*p* < 0.05) (Table 2). Notably, 23.33% of the women in the BEs group achieved an FSFI total score greater than 28.1, while none of the women in the control group reached this threshold at post-study. Additionally, the BEs intervention resulted in significantly greater increases in FSFI total scores compared to the control group (MD = 5.01, 95% CI = 3.21 to 6.79, *p* = 0.001), corresponding to a large effect size (Cohen’s d = 1.43) (Table 2). The baseline and post-intervention scores for each of the six FSFI domains are presented in Supplementary Table 1.

### Exercise capacity

At the end of the study, the mean change in the distance walked in the BEs group was 42.8 ± 60.54 m, indicating a significant within-group improvement (*p* < 0.05), compared with a mean change of 14.51 ± 57.67 m in the control group, which showed a non-significant within-group increase (*p* > 0.05) (Table 2). Comparison of the mean changes in 6MWT distance between the two groups revealed no significant difference (MD = 28.28 m, 95% CI = − 2 to 58.57, *p* = 0.06), with a small-to-moderate effect size (Cohen’s d = 0.48) (Table 2).

## Discussion

This study is the first to examine the effect of BEs on depression, sexual function, and exercise capacity in postmenopausal women. In this population, 12 weeks of BEs led to significant improvements in depression and sexual function compared to a non-exercising control group. The changes in exercise capacity were not significant between the two groups.

Depression remains a common psychiatric complaint among postmenopausal women; therefore, the BDI-II was used to assess depressive symptoms in this trial. Results showed that BEs led to significant improvements in depressive symptoms compared with the non-exercising group, with a between-group mean difference of -4.22 that exceeded the predetermined minimal clinically important difference (MCID) of the BDI-II (i.e., ≥ 3)^34^, supporting the clinical relevance of the BEs intervention. Diaphragmatic BEs may stimulate the vagus nerve, enhancing parasympathetic activity and promoting relaxation^13^, which can help counteract the heightened sympathetic tone often linked to mood disturbances in postmenopausal women^35^. Given the marked estrogen reductions during this period, which increase vulnerability to depression^36^, BEs may improve emotional regulation by influencing cortisol levels and heart rate variability^14^. Incorporating diaphragmatic breathing into daily routines may also enhance mindfulness and self-awareness^37^, fostering psychological resilience during the postmenopausal transition. Consistent with our findings, diaphragmatic BEs have been shown to reduce depressive symptoms in women with systemic sclerosis after 12 weeks^38^ and in breast cancer patients after 8 weeks^39^. Furthermore, a video-based exercise intervention incorporating breath control was recently found to significantly reduce perceived stress levels of the pre-menopausal women^40^. Considering that postmenopausal depression can adversely affect functional outcomes^41^, quality of life^42^, and overall life satisfaction^43^, the observed improvements in depressive symptoms in this study are likely of clinical relevance.

Another major concern in postmenopausal women is sexual dysfunction. All women in this trial experienced sexual dysfunction. The improvement in sexual function following BEs was significantly greater compared to the non-exercising control group, yielding a between-group difference of 5.01 that exceeded the MCID of the FSFI (i.e., 4.2)^44^. The beneficial effects of BEs on sexual function may be related to improvements across multiple FSFI domains. The crook lying position and controlled exhalation, components of the BEs used in this study, may encourage a rhythmic co-activation of the deep core muscles and the pelvic floor^45^. Slow, controlled breathing could improve pelvic floor coordination^15^, potentially alleviating pain of pelvic origin^46^. Autonomic shifts promoted by BEs may enhance peripheral blood flow^47^, which could improve vaginal lubrication and genital sensitivity^48^, both of which typically decline during the postmenopausal period^49^. Given that dyspareunia and vaginal dryness can worsen with stress and muscular tension^50^, BEs may therefore promote muscular relaxation, potentially mitigating these symptoms and supporting a more comfortable sexual experience^51^. Consistent with these mechanisms, Trudel and Saint-Laurent reported that women practicing sexual awareness and breathing techniques showed greater orgasmic improvements than those performing pelvic floor exercises alone^52^. Similarly, combining breath control with body awareness through yoga has been associated with improved overall sexual function and satisfaction^53^.

BEs led to a non-significant improvement in exercise capacity compared with the control group. This borderline non-significance (i.e., *p* = 0.06) may reflect insufficient statistical power, as the study was powered for depression rather than for detecting differences in exercise capacity. Although previous studies have reported a wide range of MCID for the six-minute walk distance (i.e., 15–80 m) across various populations^54,55^, none have specifically examined postmenopausal women within the age range of the present study. It is plausible that the observed MD of 28.28 m may indicate a clinically meaningful improvement, warranting longer-duration studies for confirmation. BEs may enhance efficient oxygen delivery to working muscles and improved aerobic metabolism^16^. Furthermore, the rhythmic co-activation of the core and pelvic floor muscles achieved through the crook lying posture and controlled breathing may optimize diaphragmatic mechanical advantage^56^, thereby improving overall respiratory efficiency^56^ and functional endurance^57^. Eight weeks of respiratory exercises led to significant improvements in treadmill performance time in older women^58^, in addition to enhancing six-minute walk distance in children with thalassemia after 12 weeks^59^.

This trial has several notable strengths. It is the first study to our knowledge to explore the effects of BEs on depression, sexual function, and exercise capacity in postmenopausal women. The multi-center recruitment design enhances the external validity of the findings. However, several limitations must be acknowledged. The absence of a physical activity control group is a key limitation of this study. Since the BEs group received more in-person contact than the control group, an attention-related improvement in mood cannot be ruled out and should be addressed in future research. In addition, the relatively short duration of the intervention (i.e., 12 weeks) may not fully capture the long-term sustainability of the observed benefits, particularly for chronic conditions such as depression and sexual dysfunction. Moreover, the lack of post-intervention follow-up further limits conclusions about the durability of effects. Furthermore, participants and exercise supervisors were not blinded to group allocation. Also, the outcomes of depressive symptoms and sexual function were self-reported, which may have been influenced by social or psychological stresses. Finally, the strict inclusion criteria for the participating women may restrict the generalizability of the findings to the broader population of postmenopausal women, particularly those with comorbidities, severe mental health conditions, or those who are sexually inactive or have less frequent sexual activity.

## Conclusion

In summary, the findings of this randomized controlled trial demonstrate significant short-term improvements in depression and sexual function among a select group of postmenopausal women with mild-to-moderate depression following BEs. These results suggest that 12 weeks of diaphragmatic BEs could serve as a viable non-pharmacological adjunct to standard treatment. Although no statistically significant change was observed in exercise capacity, the overall positive outcomes support further investigation into the long-term durability and generalizability of this intervention. Based on these findings, BEs may be considered a potential complementary approach for postmenopausal women with similar characteristics, though additional research is needed before definitive clinical recommendations can be made.

## Supplementary Information

Below is the link to the electronic supplementary material.

Supplementary Material 1

## Data Availability

The datasets analyzed during the current study are available from the corresponding author on reasonable request.
