# Supplementary material for: Effect of breathing exercises on depression, sexual function, and exercise capacity in postmenopausal women: a randomized controlled trial
Source: Sci Rep. 2026 Feb 23;16:10221. doi: 10.1038/s41598-026-41198-8 (PMC13031785; doi:10.1038/s41598-026-41198-8)
Supplement: Supplementary file 1 — Supplementary Material 1 [file 41598_2026_41198_MOESM1_ESM.pdf]

Outcome			BEs (n = 30)	Control (n = 31)
FSFI	Desire	Pre-study	2.46± 1.07	2.48± 1.02
		Post-study	3.73± 0.94*	2.96± 1.04*
	Arousal	Pre-study	2.66± 1.02	2.64± 1.11
		Post-study	3.76± 1*	2.96± 0.91
	Lubrication	Pre-study	2.43± 1.07	2.48± 1.06
		Post-study	3.76± 1.45*	3.25± 1.53*
	Orgasm	Pre-study	2.66± 1.12	2.54± 1.12
		Post-study	3.7± 0.91*	3.09± 1.22*
	Satisfaction	Pre-study	2.73± 0.9	2.51± 1.06
		Post-study	3.46± 1.16*	3± 0.89*
	Pain	Pre-study	2.53± 1.13	2.54± 1.12068
		Post-study	3.5± 1*	3.12± 1.17*

Supplemental Table S1 Measures in the domains of the FSFI. Means and standard deviations are reported; a p-value lower than 0.05 donates statistical significance. * denotes significant paired t-test test. BEs stands for breathing exercises and FSFI stands for Female Sexual Function Index.
